# Postoperative bleeding and biliary leak after liver resection: A cohort study between two different fibrin sealant patches

**DOI:** 10.1038/s41598-019-48529-y

**Published:** 2019-08-19

**Authors:** Diego López-Guerra, Jesús Santos-Naharro, Adela Rojas-Holguín, Isabel Jaen-Torrejimeno, Aranzazu Prada-Villaverde, Gerardo Blanco-Fernández

**Affiliations:** Department of HBP and liver transplant surgery, Hospital Universitario Infanta Cristina, University of Extremadura, Badajoz, Spain

**Keywords:** Medical research, Liver

## Abstract

Different topical products have been tested in liver resection to get a control of bleeding. This study compares the effectiveness and complications between two haemostatic agents Tachosil versus Hemopatch. A cohort study including patients who underwent liver resection since November 2014 to April 2016 was conducted. The study was performed in a single institution. Demographic variables, intraoperative characteristics and postoperative complications were analysed. A total of 92 patients (50 in Tachosil group and 42 in Hemopatch group) were included. No differences were found in patients who required intraoperative (Tachosil 6 (12%) vs Hemopatch 2 (4.8%); p = 0.28) and postoperative (Tachosil 4 (8%) vs Hemopatch 3 (7.1%); p = 0.87) blood transfusion. There were no differences in length of hospital stay (Tachosil 7.02 ± 4.1 days vs Hemopatch 7.63 ± 9.1; p = 0.67). Overall postoperative complications were similar between both patches (Tachosil 21 (42%) vs Hemopatch 14 (33%); p = 0.48). No differences were found in specific complications, however Hemopatch showed a higher incidence of intraabdominal abscess 5 (11.9%) and vs 0 (0%) p = 0.01.In this study no differences have been found between Hemopatch and Tachosil in the effectiveness and overall postoperative complication after liver resection, although Hemopatch shows a higher incidence of intraabdominal abscess. Further studies are necessary to confirm these findings.

## Introduction

Nowadays liver resection is an intervention with considerable rates of morbi-mortality. One of the major predictors of that is the amount of intra-operative blood loss^1–5^.

Perioperative complications have been associated with predictive factors as ASA (American Society of Anesthesiologists) score, low serum albumin, major liver resection, perioperative transfusion, prolonged perioperative time, jaundice, smoking, major biliary procedures, associated extrahepatic procedures and prolonged ischaemic time^6–12^.

In high-volume centres there are specific complications with liver resection of approximately 10–20%^13^.

New advances in surgical techniques and parenchymal transection devices have reduced postoperative complications due to a better control of bleeding and biliary fistula^6,14^.

To get a suitable control of bleeding are provided conventional methods such as suture, ligation or argon beam coagulation. Several topical products have been designed with the objetive of reducing bleeding and bile leak in postoperative course, these products are gelatin, collagen, oxidized regenerated cellulose, fibrin sealant glues and synthetic glues^15–20^.

Despite the widely use of topical haemostatic agents after liver resection, the available data are heterogeneous^18^. Several reports have tried to demonstrate the utility of these agents in liver surgery. Figueras *et al*. studied Fibrin glue sealant in a large randomized trial and showed no efficacy for reducing overall and liver specific complications^16^.

Tachosil (Takeda Austria GmbH; Linz, Austria) is a ready-to-use fixed combination of a collagen sponge coated with a fibrinogen and thrombin layer, combining the mechanical support of a collagen fleece with the haemostatic and adhesive properties of the coagulation factors I and IIa. Several reports have compared Tachosil versus different haemostatic agents.

A new haemostatic pad agent is being used after liver resection in recent years. Hemopatch (Baxter Healthcare S.A.; Zürich, Switzerland) consists of a specifically formulated collagen matrix that is derived from bovine dermis. The mechanism of action creates rapid and lasting haemostasis by sealing the bleeding surface and promoting haemostasis^21^.

In preclinical studies Hemopatch has been compared vs Tachosil, demonstrating the utility of the haemostatic and sealing effectiveness^22^.

To our knowledge there is no clinical studies comparing Hemopatch with Tachosil after liver resection. The aim of this study was to compare the safety and efficacy of Hemopatch vs Tachosil after liver resection.

## Patients and Methods

From November 2014 to April 2016, 92 consecutive patients who underwent hepatic resection and met the inclusion criteria were included in the study, which was performed in a single institution. All patients were operated by the same surgical team. The data were retrieved from a prospective database of liver resection. The inclusion criteria were: provision of informed consent, patients ≥18 years of age, elective liver resection, at least segmental resection (anatomic/nonanatomic) of the liver. The exclusion criteria were: anamnestic or clinical evidence of coagulation disorders, emergency surgery, associated extrahepatic procedures such as simultaneous colorectal resection. Patients were followed-up for at least 3 months after surgery. The study was approved by the ethical and research studies committee of Badajoz University Hospital and informed consent was obtained from all patients. All experiments were performed in accordance with relevant guidelines and regulations.

They were divided in two cohort groups: Tachosil group vs Hemopatch group. The choice if was necessary a placement of a patch was determined by the surgeon. If the surgeon considered that could exist a risk of haemorrhage or biliary leakage, then a patch was used; this point was determined by the extension of the resection, the frailty of parenchymal surface and the exposure of vessel with diameter > 3 mm. The use of one of the two patches depended of the availabity stock in the moment of the surgical procedure. Despite of that every surgeon used both patches in the different procedures. The definition of anatomical hepatectomy was following the nomenclature system of Brisbane 2000^23^. Major hepatectomy was defined as the resection of 3 or more liver segments. Parenchymal transection of the liver was performed by ultrasonic dissector (CUSA). Haemostasis and biliostasis of the cut surface were achieved with electrocautery, clips and ligatures. In some cases, portal pedicles and suprahepatic veins were sectioned by vascular stapler. Pringle maneuver was used only in selected cases when necessary and always as intermittent clamping. At the end of hepatectomy, liver surface was exhaustively reviewed for bleeding and bile leakage. When we performed a minor resection it was used one patch. In case of major resection we used two patches to cover liver surface. The size of Hemopatch is 4.5 × 9 cm and the size of Tachosil is 9.5 × 4.8 cm.

Biliary fistula was based on the postoperative findings according to the definition of the classification of International Study Group of Liver Surgery (ISGLS)^24^: Bile leakage is defined as fluid with an increased bilirubin concentration in the abdominal drain or in the intra-abdominal fluid on or after postoperative day 3, or as the need for radiologic intervention (ie, interventional drainage) because of biliary collections or relaparotomy resulting from bile peritonitis.

The grade of bile leakage was according with the classification of the ISGLS:

- Grade A: Bile leakage requiring no or little change in patients’ clinical management
- Grade B: Bile leakage requiring a change in patients clinical management (eg, additional diagnostic or interventional procedures) but manageable without relaparotomy, or a Grade A bile leakage lasting for > 1 week
- Grade C: Bile leakage requiring relaparotomy

Characteristic in drains was divided as:

- Clear fluid which is clear fluid
- Mildly hematic fluid which is a red almost transparent fluid
- Hematic fluid which is a drain with blood.

Drains were removed when a total volume after 24 hours was less than 50 ml and characteristic of it was clear or mildly hematic.

On preoperative and postoperative days 1, 3 and 5 were recorded measurements of alanine aminotransferase, aspartate aminotransferase, International normalized ratio (INR), bilirubin, haematocrit, haemoglobin and platelet cells.

Operative mortality was defined as death within 30 days after the hepatic resection or admission within the same hospital for surgery. Postoperative complications were classified as reported by Dindo *et al*.^25^.

### Statical analysis

Continuous variables were analysed using Student t test. The Fisher exact test and the Pearson chi-square test were used to analyse categorical data. P < 0.05 was considered statistically significant. The Statistical Package for the Social Sciences (SPSS, release 17.0 for Windows) was used for all analyses.

## Results

**Figure 1:**
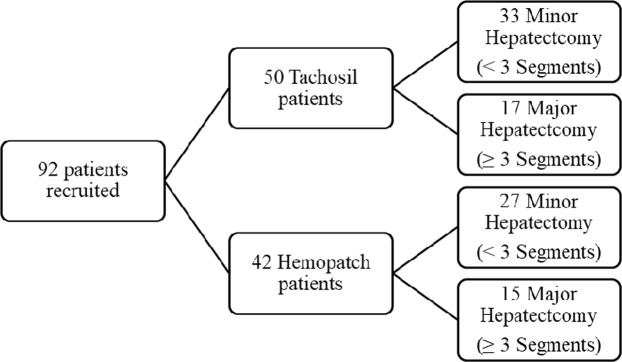
Flowchart showing distribution in both hemostatic groups.

A total of 92 patients were recruited (50 patients in Tachosil group and 42 patients in Hemopatch group). Flowchart of the groups is shown in Fig. 1. In Tachosil group 33 patients had minor hepatectomy and 17 patients had major hepatectomy. In Hemopatch group 27 and 15 patients had minor and major liver resection, respectively.

Baseline variables of patients are shown in Table 1. No differences were found in age and sex between both haemostatic groups. There were more patients with diagnosis of metastases in Tachosil group (68% vs 42.9%; P = 0.01) in contrast of a greater number of patients with diagnosis of hepatocellular carcinoma in Hemopatch cohort (23.8% vs 6%; P = 0.01). No differences were found in others diagnostics. Preoperative ASA score distribution was similar in both groups. Most of patients were ASA 2 (56% Tachosil vs 64.3% Hemopatch P = 0.42) and ASA 3 (36% vs 28.6% P = 0.45). Preoperative chemotherapy administration was higher in Tachosil cohort (64% vs 38.1% P = 0.01).

**Table 1:** General Characteristics of patients.

Characteristics	Tachosil (n = 50)	Hemopatch (n = 42)	P Value
Age, mean (SD*), y	62.4 (13.4)	61.4 (14.6)	0.71
Sex, male:female	28:22	28:14	0.29
ASA 1/2/3/4/5	2/28/18/2/0	3/27/12/0/0	0,43
Preoperative Chemotherapy	32 (64)	16 (38,1)	0,01
**Diagnosis for resection**			
Metastases No. (%)	34 (68)	18 (42.9)	0.01
Hepatocellular Carcinoma No. (%)	3 (6)	10 (23.8)	0.01
Cholangiocarcinoma No. (%)	1 (2)	2 (4.8)	0.59
Bening Diseases No. (%)	8 (16)	6 (14.3)	0.82
Others No. (%)	4 (8)	6 (14.3)	0.50

*Standard deviation.

Intraoperative data are shown in Table 2. No differences in liver parenchyma characteristics were found with similar absence of steatosis (54% vs 50% P = 0.70) and steatosis presence (44% vs 35.7% P = 0.42), however there were more patients with cirrhosis in liver parenchyma in Hemopatch group (2% vs 14.3% P = 0.04). No differences were found in the percentage of patients requiring intraoperative transfusion in the two groups (Tachosil 6 (12%) vs Hemopatch 2 (4.8%)) but there was an increase in mean (SD) packed read blood cell in group of Tachosil 2 (0.63) vs Hemopatch 0.75 (0.95) (P = 0.03). Patients who required Pringle clamping maneuver was similar in both groups (Tachosil 12 (24%) vs Hemopatch 11 (26.2%); P = 0.80) and mean time (SD) also showed no differences with 27 minutes (14) in Tachosil group and 24 min (19) in Hemopatch group (P = 0.68). Operating time mean (SD) was slightly higher in Tachosil group 202 minutes (69) vs Hemopatch group 184 min (55), but these differences were no statistically significant P = 0.16.

**Table 2:** Intraoperative data of patients in Tachosil and Hemopatch group.

Intraoperative data	Tachosil (n = 50)	Hemopatch (n = 42)	P Value
Intraoperative blood transfusión N° (%)	6 (12)	2 (4.8)	0.28
Intraoperative Blood Transfusion of PRBC^a^ units, mean (SD^*^)	2 (0.63)	0.75 (0.95)	0.03
**Liver parenchyma**			
No Steatosis, No. (%)	27 (54)	21 (50)	0.70
Steatosis, No. (%)	22 (44)	15 (35.7)	0.42
Cirrhosis, No. (%)	1 (2)	6 (14.3)	0.04
Patients with Plingle clampling N°. (%)	12 (24)	11 (26.2)	0.80
Clampling time, mean (SD), min	27 (14)	24 (19)	0.68
Operating time, mean (SD), min	202 (69)	184 (55)	0.16

^a^packed red blood cell.

^*^Standard deviation.

Postoperative data are shown in Table 3. Surgical drainage was maintained a mean (SD) of 4.4 (2.6) days in Tachosil group and 4.1 (1.9) days in Hemopatch group (P = 0.67). There was not any difference in fluid discharge quality between both groups: Clear fluid: 40.4% in Tachosil group and 43.6% in Hemopatch group; mildly hematic fluid 51.1% vs 46.2% and hematic fluid 8.5% vs 10.3%. The mean (SD) length of hospital stay was 7.02 (4.1) days in Tachosil group and 7.63 (9.1) days in Hemopatch group (P = 0.67). During their postoperative hospital stays, 4 patients of Tachosil group (8%) received blood transfusion and in Hemopatch group 3 (7.1%) of them. None patient received fresh frozen plasma transfusion neither platelet transfusion in both groups.

**Table 3:** Postoperative data of patients in Tachosil and Hemopatch group.

Postoperative data	Tachosil (n = 50)	Hemopatch (n = 42)	P Value
Length of Hospital Stay, mean (SD^*^), days	7,02 (4,1)	7,63 (9,1)	0,67
Drainage day Remove, mean (SD)	4,4 (2,6)	4,1 (1,9)	0,54
Postoperative blood transfusion No. (%)	4 (8)	3 (7,1)	0,87
Postoperative FFP^a^ transfusion No. (%)	0 (0)	0 (0)	—
Postoperative Platelet transfusion No. (%)	0 (0)	0 (0)	—

^a^fresh frozen plasma.

*Standard deviation.

**Figure 2:**
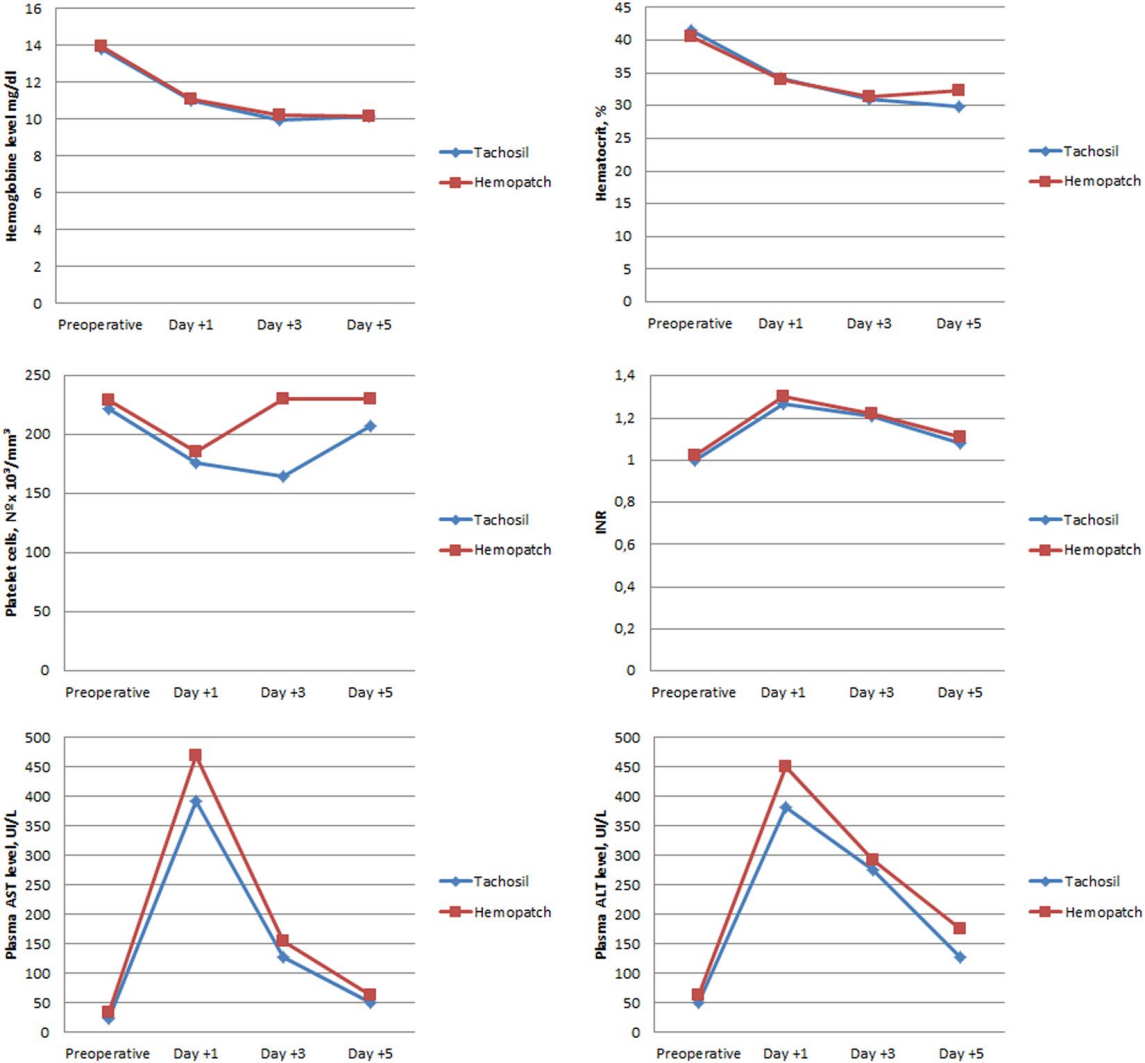
Postoperative biochemical parameters at registered at day 1, 3 and 5. Values are mean in each postoperative day.

No differences were found in postoperative biochemical parameters between two groups (Fig. 2). Postoperative parameters were registered at postoperative day 1, 3 and 5.

Postoperative complications occurred in 21 (42%) patient in Tachosil group and 14 (33%) patients in Hemopatch group (Table 4). The analyse severity of complications showed no differences between both groups, most of them were grade I (66,7% Tachosil group vs 57,1% Hemopatch group; P = 0.31). A higher rate of grade III complications were found in Hemopatch group 28,6% versus 4,8% in Tachosil group (P = 0.17). One postoperative death occurred in both groups. The analyse of specific complications showed no differences between both groups in haemorrhage which no occurred in any patient, bile leakage (2% in Tachosil group vs 4.8% in Hemopatch group; P = 0.46) all of them were biliary fistula grade A; and reoperation (2% in Tachosil group vs 2,4% in Hemopatch group; P = 0.90). However Hemopatch showed a higher incidence of intraabdominal abscess 5 patients (11.9%) and none patient in Tachosil group p = 0.01. A multivariable analysis was performed to identify independent covariates that predict postoperative intraabdominal abscess; none of the variables studied included fibrin sealant patches were found to be independent predictors of that complication.

**Table 4:** Postoperative complications of patients in Tachosil and Hemopatch group.

Postoperative Complications	Tachosil (n = 50)	Hemopatch (n = 42)	P Value
Total Complications No. (%)	21 (42)	14 (33,3)	0,48
Grade I No. (%)	14 (66,7)	8 (57,1)	0,31
Grade II No. (%)	5 (23,8)	1 (7,1)	0,21
Grade III No. (%)	1 (4,8)	4 (28,6)	0,17
Grade IV No. (%)	0 (0)	0 (0)	—
Grade V No. (%)	1 (4,8)	1 (7,1)	>0,99
Hemorrhage No. (%)	0 (0)	0 (0)	—
Bile Leakage No. (%)	1 (2)	2 (4,8)	0,46
Reoperation No. (%)	1 (2)	1 (2,4)	0,90
Intraabdominal Abscess No. (%)	0 (0)	5 (11,9)	0,01
Readmission No. (%)	0 (0)	4 (9,5)	0,02

## Discussion

In this study we compare two different fibrin sealant patches trying to determine if there are differences in effectiveness and postoperative complications. Tachosil has been used in several studies after liver resection showing benefits in terms of a better control bleeding.

A prospective study by Briceno *et al*. found an advantage for Tachosil compare with a control group reducing drain output, postoperative transfusion requirements and moderate to severe postoperative complications in major liver resections^26^.

Frilling *et al*. studied Tachosil and reported shorter time to haemostasis vs argon beamer^27^.

Zacharias *et al*. compared Tachosil vs Surgicel after liver resections and they didn’t find reduce overall complication rate, but similar to other studies suggest that Tachosil may be beneficial when performing a major hepatectomy^28^.

Recent studies as Weltert L *et al*.^29^ has shown that the use of Hemopatch is effective in stopping bleeding in patients undergoing ascending aorta cardiac procedures. Fingerhut *et al*.^30^ described a series of case reports in which Hemopatch is useful in different surgical procedures to control bleeding.

In our study no differences were found in the blood transfusion requirement between the both groups. None haemorrhage occurred in any patient thus showing equal haemostatic effectiveness of both patches. We have also found no differences in bile leakage suggesting a possible effect that would prevent this complication even though Hemopatch is not approved as sealant. One of the limitations of our study is that there was no randomization, is an observational study which could cause bias in the results. The decision to place a patch depends of subjective factors so it could be a limitation of our study. Another limitation of the study is the number of patients which adding higher number could increase the statistical power. However the low grade of complications in both groups, above all, biliary leakage and haemorrhage probably would not change the major endpoints of the study.

Despite biliary leakage has been associated with an increase rate depend of a specific operative procedure^31^, in our study only three patients developed biliary leakage, we did not find any relationship between biliary leakage and a specific operative procedure. None of the three patients had intraoperative biliary leakage but in all of them a drainage was placed; in some studies as T. Ishii *et al*. a placement of a drainage has been recommended for preventing biliary fistula. In case of post-operative bleeding none patient developed this complication so there was no relationship with the operative procedure.

Although in the Tachosil group the overall percentage of complications was slightly higher, grade III complications rate in the Hemopatch group were greater. Intraabdominal abscess was more frequent in Hemopatch group with statistically significant differences, and also was associated with more readmissions in this group.

Higher intraabdominal abscess rates in this group could be explained because there is more patients with cirrhosis in this group. It is known that cirrhosis is associated with immune dysfunction which involves a state of immunodeficiency, and in parallel a state of persistent activation of the immune system cells with production of pro-inflammatory cytokines^32^. Innate and adaptive immune system are affected, cirrhosis leads to reduced numbers of circulating immune system cells^33^. Also, Cirrhosis results in reticuloendothelial dysfunction, due to reduced number of liver reticuloendothelial mononuclear cells in liver and porto-systemic shunting, and there is a decrease hepatic synthesis of molecules of the innate immune response^34^. In addition exist an induced expression of activation molecules on the surface of immune cells and the increased synthesis of pro-inflammatory cytokines, especially by monocytes^35^.

In terms of haemostatic effectiveness, we found no differences between the two groups with similar postoperative blood transfusion rates and in the evolution of postoperative biochemical parameters. Length of hospital stay is equal in both groups as well as drainage day remove.

As far as we know, a comparative study in human patients between these two haemostatic patches has not been done yet. These results suggest that Hemopatch is not inferior to Tachosil becoming a good option to use as topical haemostatic after liver resection. Although we have achieved two reasonably well-balanced groups, not being a randomized trial is a limitation of the study. In the other hand the greater number of cirrhotic liver resection in one of the arms which could result in skewed results and thus, our conclusions strength must be tempered.

## Conclusion

No differences have been found between both patches in haemostatic effectiveness, however Hemopatch shows higher rate of intraabdominal abscess and readmission. More studies are necessary to verify these results.
